# Case of Œsophagotomy

**Published:** 1855-02

**Authors:** 


*Case of Œsophagotomy.*—Singho Naide, a native of Colombo, aged 40, a fisherman by occupation, was taken into the Pettah Hospital on the evening of the 12th of August, 1853, a fish, which he held between his teeth while bating a hook, having slipped back into, and remained impacted in, the œsophagus. On examining the neck, it appeared swollen, with a feeling as if there were fluid in the cellular tissue about the muscles of the neck. In the fauces the tail of the fish was felt, and could be seen distinctly on pressing the tongue. The tail was inclined toward the left side of the throat, showing the direction the fish had taken in its course down the œsophagus. Careful examination externally failed in discovering the situation of the fish, and it was found impracticable to withdraw it from the throat, for reasons which will appear obvious when the fish is described. An incision was made between the anterior edge of the sterno-mastoid muscle and the trachea, commencing at the lower edge of the os hyoides, and extending down to the sternum. After a most diligent search, both by myself and my friends, nothing was discovered to indicate the spot where the gullet should be divided. The next step of the operation was conducted with great care. The passing of a male catheter was entrusted to Dr. Elliott, who, with no little difficulty, introduced it into the gullet, directed by his fingers, and turned the convex side of it toward the wound. This enabled the part to be seized with a pair of forceps, and a small opening to be made into the œsophagus. The finger introduced into this opening gave the feeling of something cartilaginous being lodged, which was soon found to be the edge of the fish. A polypus forceps was introduced, and attempts were made to extract it, but to no purpose, as the head of the fish was too smooth to be grasped by a polished instrument. A little manœuvre with the index finger, however, soon dislodged the fish, which made its exit through the wound head foremost. The fish was four inches and a-half long from head to tail, and one inch and a-half broad. It is named by Mr. Gray, in his "Illustrations of Indian Zoology," "Anabas Spinosus," and has long and sharp fins, both on the back and near the gills. About a week after the operation, a little nourishment was given, through the mouth, but as some of it flowed out through the wound, it was deemed prudent not to repeat the attempt, but to continue nutriment through the rectum. In three or four days more the man was able to take nourishment by the mouth, from which time he began to gain flesh and strength. The wound healed gradually, and he was discharged quite cured on the 23d of September, with merely a line of cicatrix on the side of the neck. The performance of the operation occupied more than an hour, and by lamplight. —*Mr. Anthoniesz in Ceylon Miscellany and Lancet.*

